# Upregulation of CYP2S1 by oxaliplatin is associated with p53 status in colorectal cancer cell lines

**DOI:** 10.1038/srep33078

**Published:** 2016-09-09

**Authors:** Chao Yang, Qian Zhou, Minle Li, Xuemei Tong, Jiayi Sun, Yin Qing, Liya Sun, Xuhan Yang, Xiaowen Hu, Jie Jiang, Xiaomei Yan, Lin He, Chunling Wan

**Affiliations:** 1 Bio-X Institutes, Key Laboratory for the Genetics of Developmental and Neuropsychiatric Disorders (Ministry of Education), Shanghai Jiao Tong University, Shanghai 200030, China; 2 College of Life Science, Anhui Normal University, Anhui Wuhu 241000, China; 3 Department of Biochemistry and Molecular Cell Biology, Institute of Medical Science, Shanghai Jiao Tong University School of Medicine, Shanghai 200025, China; 4 School of Life Sciences & Biotechnology, Shanghai JiaoTong University, Shanghai 200240, China

## Abstract

Oxaliplatin displays a wide spectrum of antitumor activities and is widely used in the treatment of metastatic colorectal cancer (CRC). However, tumor responses to this agent are variable, and the underlying mechanisms are poorly understood. In the present study, oxaliplatin was found to strongly inhibit the growth of HCT116 cells harboring wild-type p53 but to only weakly inhibit SW480 cells, HT29 cells or p53−/− HCT116 cells, which all lack p53 expression. Administration of oxaliplatin significantly induced p53 accumulation and enhanced expression of CYP2S1 in HCT116 cells with wild-type p53. CYP2S1 knockdown conferred a cell survival advantage after oxaliplatin treatment to cells harboring wild-type p53 *in vitro* and *in vivo*. Interestingly, enzyme immunoassays, TOPFlash/FOPFlash reporter activity assays and western blotting analysis demonstrated oxaliplatin-mediated downregulation of PGE2 and Wnt/β-catenin signaling in a manner dependent on p53. Moreover, oxaliplatin treatment of mice with subcutaneous tumor xenografts drastically reduced the volume of wild-type p53 HCT116 tumors but had no effect on isogenic p53−/− HCT116 tumors. These results suggest that oxaliplatin exerts its inhibitory effects in human CRC cells via upregulation of CYP2S1 expression in a p53-dependent manner.

Colorectal cancer (CRC) remains a major public health problem for both men and women, the disease is estimated to affect more than 1 million people worldwide and to cause approximately half a million deaths annually^1^,^2^. Although the mainstay of potentially curative treatments for CRC is surgical resection, prognosis is generally poor due to locoregional recurrence with resection alone^3^. Therefore, the FDA has recommended chemotherapy as the primary treatment for advanced CRC.

Oxaliplatin (Eloxatin^®^, OXA), a third-generation chemotherapy drug of the diaminocyclohexane platinum family, has potent *in vitro* cytotoxicity and *in vivo* antitumor activity. Indeed, cisplatin-resistant colorectal tumors are responsive to oxaliplatin^4^. In advanced colorectal carcinoma, oxaliplatin produces response rates of 2 to 24% in untreated patients and approximately 10% in patients who have relapsed or are refractory to treatment^5^. Oxaliplatin induces the formation of DNA adducts and interstrand cross-links owing to the restricted freedom of movement of the platinum atom, thus impeding DNA replication and transcription^6^. Oxaliplatin causes cell-cycle arrest, promotes accelerated senescence and induces apoptosis in cancer cells^7^,^8^,^9^.

The p53 protein is involved in many biological processes, the best known of which are cell-cycle arrest and DNA repair^10^,^11^. p53 also regulates apoptosis after exposure to hypoxia and cytotoxic drugs and is one of the most commonly mutated genes in many types of cancer^12^. Oxaliplatin treatment upregulates p53, and activated p53 enhances growth inhibition in CRC cells treated with oxaliplatin. In contrast, silencing p53 significantly decreases the inhibitory effects of oxaliplatin, suggesting an important role for p53 in this process^13^,^14^. The p53 protein regulates a group of cytochrome P450 (CYP) genes in human and mouse liver cells and influences the efficacy of chemotherapeutic treatment regimens^15^,^16^. However, a role for p53 in regulating CYP450 genes in the intestinal tract has not yet been reported.

CYP450 enzymes play a major role in the oxidative metabolism of numerous endogenous and exogenous compounds (including pharmacological drugs) and thus are a primary defense against these compounds^17^,^18^. Increased expression of specific CYP proteins is a key component of this defense^19^. For example, CYP2S1, which is most highly expressed in intestinal tract epithelial cells, may be involved in metabolizing aromatic hydrocarbons and other xenobiotic substrates^20^,^21^. Madanayake *et al.*^22^ initially showed that depletion of CYP2S1 enhanced bronchial epithelial cell proliferation most likely through PGE2 synthesis^22^. Bui *et al.* also identified that human CYP2S1 is an important enzyme in the metabolism of COX-derived prostaglandins at nanomolar concentrations, and the authors suggested that CYP2S1 may play an important role in modulating the inflammatory process^23^. As a promising chemotherapeutic agent for treatment of CRCs, the half-life of oxaliplatin in the body is approximately 40 hours, and its metabolism may influence its efficacy. Recently, RNA-seq data analysis suggested that Wnt/β-catenin signaling and cytochromeP450 enzymes (CYP51A1) were correlated to oxaliplatin sensitivity in 21 colorectal cancer cell lines^24^. We previously demonstrated that CYP2S1 is regulated PGE2-mediated activation of β-catenin signaling and influences CRC cell proliferation *in vitro* and *in vivo*^25^. Therefore, the characterization of CYP2S1 in the process of influencing oxaliplatin activity may lead to potential strategies for colorectal cancer treatment.

β-catenin is a crucial signaling molecule in the Wnt pathway and plays important roles in cell survival and proliferation^26^,^27^. Recent studies have suggested that aberrant activation of β-catenin signaling often occurs in the early stages of human malignancies, including CRC^28^,^29^. The proposed mechanism of β-catenin associated tumorigenesis has been suggested to involve nuclear accumulation of β-catenin and interaction of β-catenin with transcription factors that control the cell cycle (such as Tcf/Lef-responsive proteins)^26^,^30^. Studies also have indicated crosstalk between β-catenin and the p53 protein^31^,^32^. Thus, β-catenin and its downstream target genes represent ideal targets for tumor therapeutics^33^.

In this study, we investigated the antitumor efficacy and mechanisms of action of oxaliplatin using *in vitro* experiments in CRC cell lines and an *in vivo* tumor xenograft model. This study is the first to report that inhibition of oxaliplatin-induced cell growth may be dependent on p53 and may involve increased expression of cytochrome enzymes (CYP2S1) in CRC cells. We also observed that oxaliplatin treatment affects intracellular PGE2 production and Wnt/β-catenin signaling. Our experiments confirm and extend the involvement of CYP2S1 as a potential therapeutic target for enhancing oxaliplatin efficacy in colorectal epithelial cells.

## Results

### Inhibition of CRC cell growth by oxaliplatin is associated with the presence of wild-type p53

**Figure 1:**
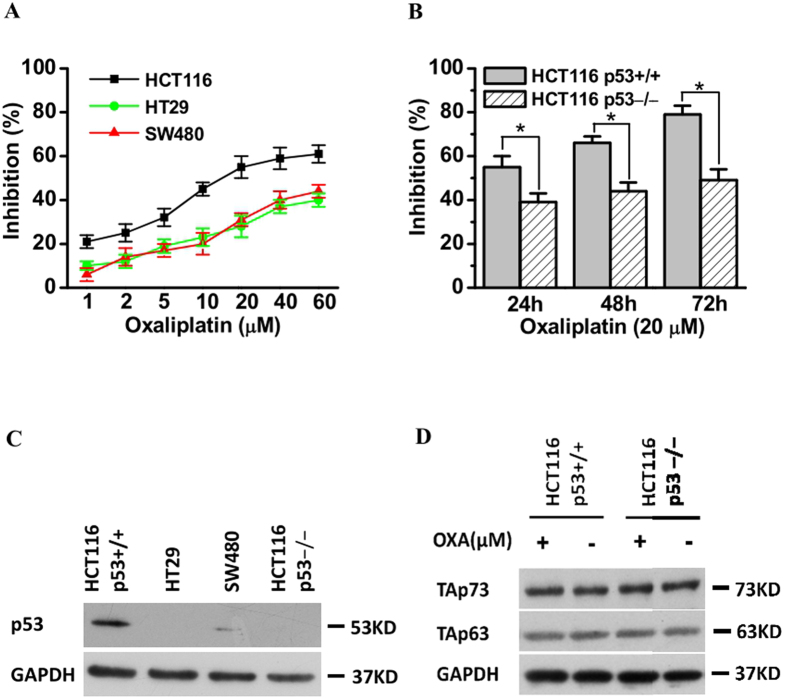
Inhibition of colorectal cancer cell growth by oxaliplatin. (**A**) Growth inhibition of 3 colorectal cancer cell lines, as detected by the CCK8 assay. HCT116(wild-type p53), HT29, and SW480 cells were treated with different concentrations of oxaliplatin for 24 h; a CCK8 assay was used to detect inhibition of cell growth as described in Materials and Methods. The rate of growth inhibition was higher in HCT116 cells than in HT29 or SW480 cells (p < 0.05). Data are expressed as the means ± SD of three independent experiments. **(B**) Isogenic p53+/+ HCT116 (wild-type p53) and HCT116 cells in which p53 was stably knocked down (p53−/− cells) were treated with 20 μM oxaliplatin for 24–72 h. A CCK8 assay was used to detect cell growth inhibition (*p < 0.05). Data are expressed as the means ± SD of three independent experiments. **(C)** Cells were treated as described in A and B, and p53 was detected in cell lysates by western blotting. The results shown are representative of three experiments. (**D**) p53+/+HCT116 cells and p53−/− HCT116 cells were treated with or without oxaliplatin (20 μM) for 24 h; total protein was extracted, and the protein levels of total TAp63 and TAp73 were analyzed by western blotting. The results shown are representative of three experiments.

To investigate the cytotoxicity of the anticancer agent oxaliplatin in CRC cells, CCK8 assays were performed using HCT116, SW480, and HT29 cells treated with various concentrations of oxaliplatin for 24 h. As shown in Fig. 1A, oxaliplatin inhibited cell growth in these three CRC cell lines in a dose-dependent manner, with HCT116 cells being more sensitive to oxaliplatin than SW480/HT29 cells (Fig. 1A). In addition, p53 expression was high in HCT116 cells and low in SW480/HT29 cells (Fig. 1C).

Next, we used isogenic p53+/+ and p53−/−HCT116 cell lines, which differ only in their p53 status, to determine whether p53 is required for chemotherapy-induced inhibition of tumor cell growth. Oxaliplatin-induced inhibition of cell growth was markedly lower in p53−/− HCT116 cells than in p53+/+ HCT116 cells (Fig. 1B,C). We know that p53 belongs to a family of related proteins, including p63 and p73. Unlike p53, p63 and p73 are not frequently mutated in human cancers. The p63 and p73 protein was also reported to be involved in cancer, apoptosis and chemosensitivity^34^,^35^. To ascertain whether less sensitivity of p53-deficient cells to oxaliplatin could be mediated through other p53 family members, we investigated the p63 and p73 proteins expression in HCT116 cells treated with or without oxaliplatin. No significant change was found in both cell lines (Fig. 1D). Together, these results demonstrate that inhibition of oxaliplatin-induced cell growth is associated with the presence of wild-type p53.

### Upregulation of CYP2S1 expression by oxaliplatin is required for p53 expression in CRC cells

**Figure 2:**
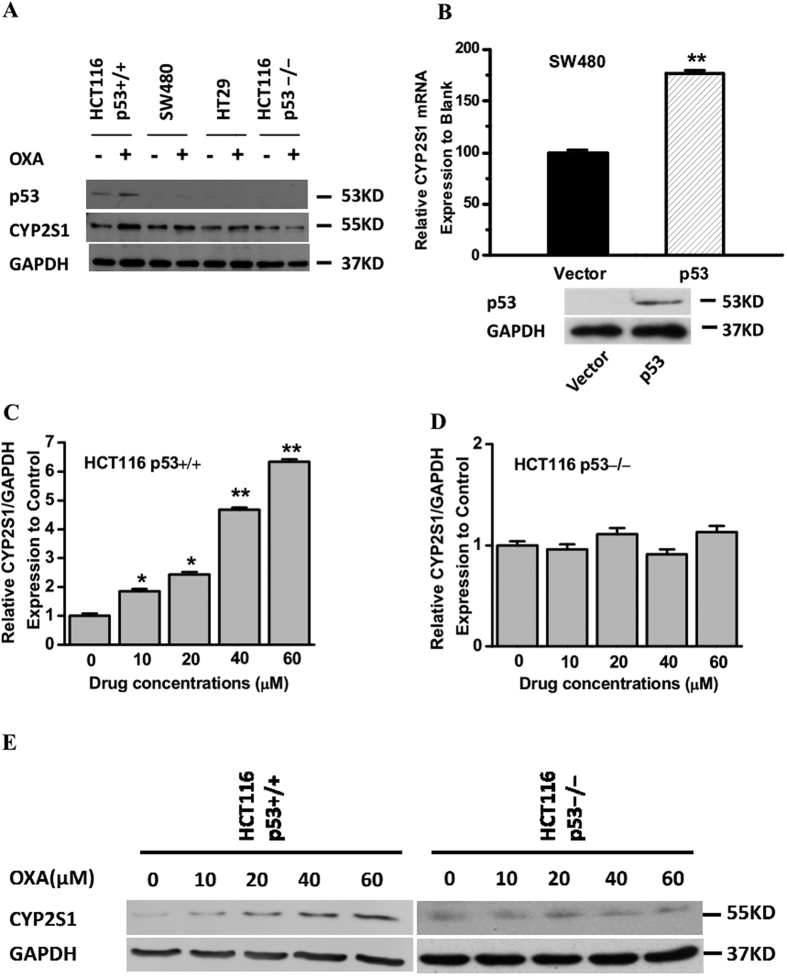
Involvement of p53 in CYP2S1 induction by oxaliplatin in colorectal cancer cells. **(A)** p53+/+HCT116 cells, SW480 cells, HT29 cells and p53−/− HCT116 cells were treated with oxaliplatin (20 μM) for 24 h; total protein was extracted, and the protein levels of total p53 and CYP2S1 were analyzed by western blotting. The results shown are representative of three experiments. **(B**) Ectopic expression of p53 markedly increased SW480 cells’ CYP2S1 transcriptional activity after Oxaliplatin treatment for 24 h. Data are represented as mean ± s.d., **p < 0.01. **(C, D)** Effect of oxaliplatin treated on CYP2S1 mRNA expression. Isogenic p53+/+ HCT116 and p53−/− HCT116 cells were treated with oxaliplatin for 24 h; total RNA was extracted, and CYP2S1 mRNA expression was analyzed by Taqman-based real-time RT-PCR analysis. GAPDH was used as an endogenous control. **(E**) Cells were treated as described in panels C and D. CYP2S1 protein expression was analyzed by western blotting. GAPDH was used as an endogenous control. Representative results from three experiments are shown.

The anticancer chemotherapeutic compound oxaliplatin, which activates p53, is metabolized by drug-metabolizing enzymes. To determine whether oxaliplatin treatment induces CYP2S1 expression, we treated colorectal cancer cell lines with an oxaliplatin dose that reflects the peak plasma concentration reported in clinical 24 h-measurements^36^. Western blots were then performed to analyze p53 and CYP2S1 protein expression. As expected, oxaliplatin treatment of p53+/+ HCT116 cells resulted in a remarkable elevation in p53 protein levels, and these cells showed an increase in CYP2S1 protein expression. Conversely, no effect was observed in p53-deficient cells (Fig. 2A). Thus, oxaliplatin induces CYP2S1 expression in CRC cells in a p53-dependent manner.

In addition, we investigated the effect of p53 ectopic expression on the activity of CYP2S1 by comparing the effects of oxaliplatin treatment in SW480 cells transiently transfecting the wild-type p53 plasmids or empty vector. Ectopic expression markedly increased the p53 level after Oxaliplatin treatment (Fig. 2B). Our results also indicated that oxaliplatin treatment increased CYP2S1 transcriptional activity in SW480 cells overexpressing p53 but not in empty vector controls (Fig. 2B).

To further verify that the induction of CYP2S1 by oxaliplatin correlates with p53 status, we treated isogenic p53+/+ and p53−/− HCT116 cells with a gradient of oxaliplatin concentrations for 24 h (Fig. 2C–E). Real-time RT-PCR analysis demonstrated strong induction of CYP2S1 mRNA by oxaliplatin in p53+/+ HCT116 cells at all concentrations, whereas oxaliplatin had no effect on p53−/− HCT116 cells (Fig. 2C,D). The results of western blotting were consistent with the above results (Fig. 2E). Taken together, these results suggest that p53 expression is involved in the induction of CYP2S1 by oxaliplatin.

### CYP2S1 knockdown conferred a cell survival advantage after oxaliplatin treatment to cells harboring wild-type p53

**Figure 3:**
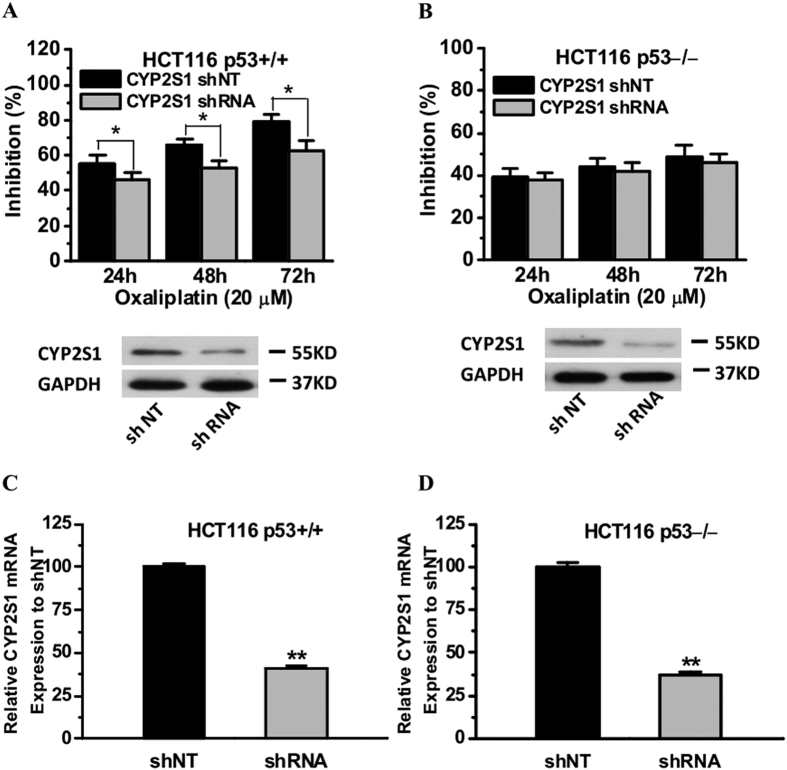
CYP2S1 knockdown elevate resistance of p53+/+HCT116 cells to oxaliplatin treatment *in vitro*. CYP2S1 knockdown conferred cell survival advantage after oxaliplatin treatment to cells harboring wild-type p53 but not to isogenic cells lacking p53. Cells were treated with the shNT (denotes non-targeting control shRNA) or CYP2S1 shRNA, respectively. Cell viability after Oxaliplatin (20 μM) treatment was determined by the CCK8 assay. **(A,B**) p53+/+ HCT116 CYP2S1 knockdown cells are highly resistant to oxaliplatin treatment. Reduced CYP2S1 protein expression in cells was confirmed by Western blot, one of three representative experiments is shown. **(C,D**) Total RNA was extracted and the mRNA levels of the CYP2S1 gene were analyzed in qRT–PCR analysis. Results are of the average from three experiments. Data are represented as mean ± s.d., *p < 0.05, **p < 0.01.

Considering that CYP2S1 expression is associated with pharmacological drug metabolism in CRC cancer. ShRNA plasmids (genechem NM_030622) was used to knockdown CYP2S1 mRNA and protein and examined whether CYP2S1 expression alters cell viability upon oxaliplatin treatment. To validate the knockdown efficiency, CYP2S1 protein and mRNA levels were measured by Western blot analysis and by quantitative real-time PCR, respectively. The results indicated that CYP2S1 mRNA and protein were clearly reduced (Fig. 3A–D). CYP2S1 knockdown led to a significant cell survival advantage after oxaliplatin treatment (20 μM, 24–72 h) in p53+/+ HCT116 cells but not in isogenic p53−/− HCT116 cells (Fig. 3A,B). The findings indicate that p53 may be potentially important for the CYP2S1effect to inhibit cell survival after oxaliplatin treatment.

### The effects of oxaliplatin on PGE2 production and Wnt/β-catenin signaling are correlated with p53 status

**Figure 4:**
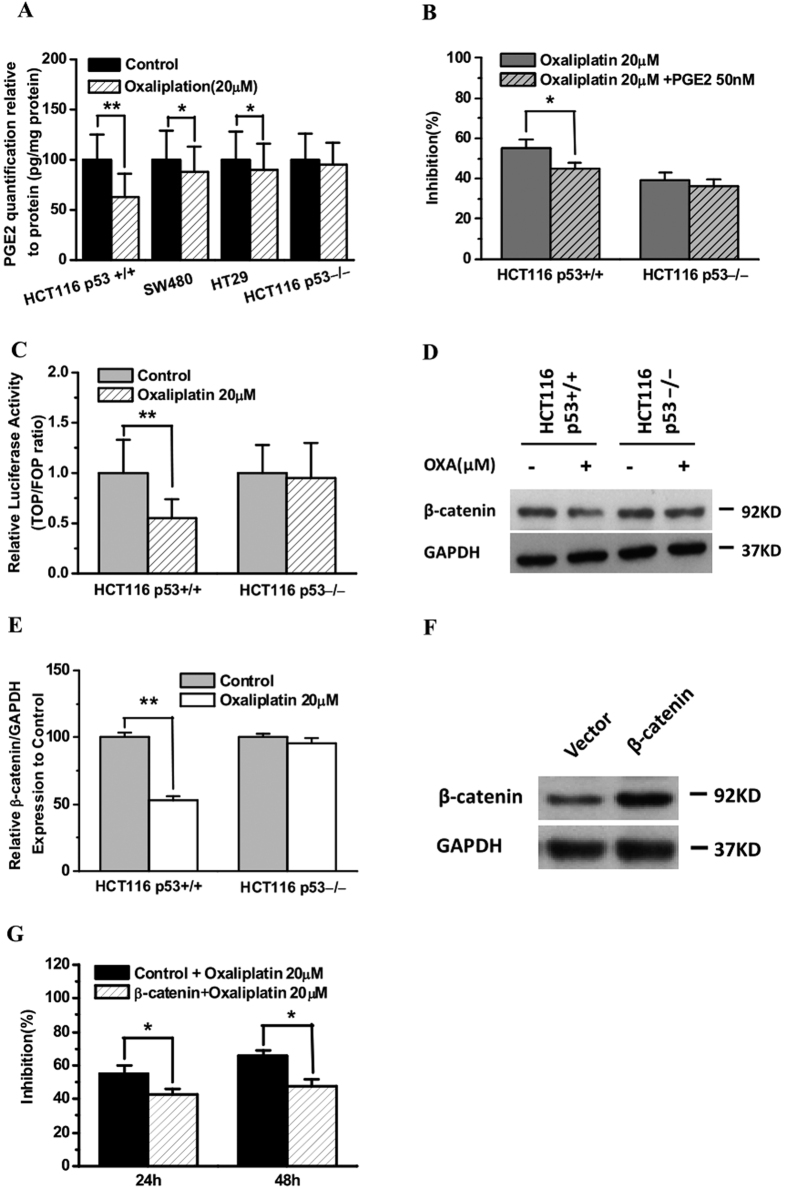
Effect of oxaliplatin on PGE2 production and Wnt/β-catenin signaling and correlation with p53 status. **(A)** Enzyme-linked immunosorbent assay of PGE2 levels. PGE2 levels in cell culture supernatants were examined after oxaliplatin (OXA) treatment, and the results are expressed in terms of pg/mg protein. The error bars indicate the variability among wells, n = 3. *p < 0.05, **p < 0.01. Data analysis was performed using Student’s *t*-test. **(B**) Treatment of cells with or without exogenous PGE2 influence oxaliplatin treatment in p53+/+ and p53−/− HCT116 cells. The data on cell growth was performed using CCK8 assay from three experiments, data analysis was performed using student’s *t*-test. *p < 0.05. **(C)** Luciferase activity assay shows that oxaliplatin treatment decreases the transcriptional activity of TCF/LEF in p53+/+ HCT116 cells. TOPFlash and FOPFlash activities were evaluated in p53+/+ HCT116 and p53−/− HCT116 cells 24 h after oxaliplatin treatment. The data are expressed as the TOP/FOP ratio relative to the control. The results represent the mean ± SD from three independent experiments. **p < 0.01. **(D)** p53+/+ HCT116 and p53−/− HCT116 cells were treated with or without oxaliplatin for 24 h, and the expression levels of β-catenin or GAPDH (internal control) were analyzed by western blotting. One of three representative experiments is shown. **(E)** Total proteins were extracted and the protein levels of the β-catenin were analyzed in Western blot analysis. Results are of the average from three experiments. Data are represented as mean ± s.d., **p < 0.01. **(F, G)** The overexpression of β-catenin conferred cell survival advantage after oxaliplatin treatment in p53+/+ HCT116 cells. Cell viability after oxaliplatin treatment (24 h or 48 h) was determined using the CCK8 assay. Increased β-catenin protein expression in cells transfected with β-catenin was confirmed by Western blotting. One of three representative experiments is shown.

CYP2S1 depletion enhances CRC cell proliferation and is associated with PGE2-mediated activation of β-catenin signaling, and p53 downregulates Wnt signaling^25^,^37^. We therefore examined whether p53 plays a role in oxaliplatin mediated regulation of CYP2S1-PGE2-Wnt signaling. CRC cells were treated with or without oxaliplatin for 24 h, and the level of PGE2 in the culture supernatants was examined by competitive ELISA. Treatment with oxaliplatin decreased the production of PGE2 in all cell lines (Fig. 4A). A significant decrease in PGE2 synthesis was observed in p53+/+ HCT116 cells, whereas only a slight decrease in PGE2 production was observed in p53-deficient cells (SW480, HT29 and p53−/− HCT116 cells) (Fig. 4A). Next, we examined whether exogenous PGE2 could affect cell growth in the processing of oxaliplatin treatment. For this purpose, Cells were treated with or without 50 nM PGE2 and 20 μM oxaliplatin for 24 h and then cell growth were determined. We found that the treatment of cells with exogenous PGE2 resulted in a significant decrease effect of inhibiting cell growth after oxaliplatin treatment in p53+/+ HCT116 (p < 0.05) (Fig. 4B).

To assess the impact of oxaliplatin treatment on Wnt/β-catenin signaling, we quantified TOPFlash activity in isogenic p53+/+ and p53−/− HCT116 cells treated with or without oxaliplatin for 24 h. The absence of p53 expression in p53−/− HCT116 cells was confirmed by western blotting (Fig. 1C). Oxaliplatin at a concentration of 20 μM resulted in reduced TOPFlash/FOPFlash luciferase activity in p53+/+ HCT116 cells, whereas no significant difference in activity was observed in p53−/− HCT116 cells (Fig. 4C). Furthermore, oxaliplatin treatment resulted in a greater reduction of β-catenin expression in p53+/+ HCT116 cells than in p53−/− HCT116 cells (Fig. 4D,E). Considering that the functional effect of β-catenin expression maybe provides a survival advantage to colorectal cancer cells in oxaliplatin treatment, we developed p53+/+ HCT116 cells transiently transfecting the β-catenin plasmids or empty vector and examined whether β-catenin overexpression alters cell viability upon oxaliplatin treatment (20 μM, 24 h or 48 h). Our results indicated that β-catenin overexpression led to a significant cell survival advantage after oxaliplatin treatment (Fig. 4F,G). Overall, these results demonstrate that oxaliplatin downregulates PGE2 production and Wnt/β-catenin signaling in a p53-dependent manner.

### Upregulation of CYP2S1 protein expression increases oxaliplatin sensitivity and is associated with p53 *in vivo*

**Figure 5:**
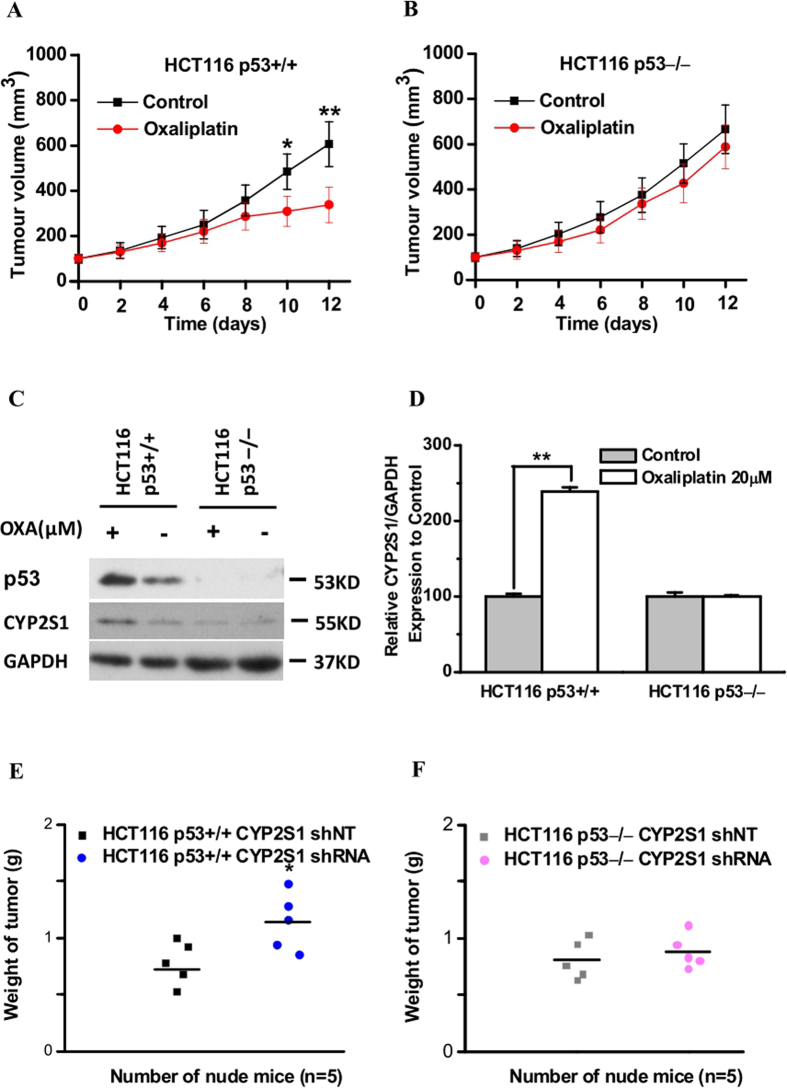
Upregulation of CYP2S1 protein expression increases oxaliplatin sensitivity in a p53-dependent manner *in vivo*. Identical (2 × 10^6^) amounts of each cells were injected subcutaneously into the flanks of nude mice. When tumors reached approximately 100 mm^3^, the mice received either PBS or oxaliplatin (10 mg/kg) intraperitoneally (day 0). A second dose of either PBS or oxaliplatin was administered three days later. Tumor growth was analyzed by caliper measurements every 2 days. **(A,B)** Comparison of tumor volume between each group (means ± SD, n = 5). Oxaliplatin treatment markedly reduced tumor volume in p53+/+HCT116 tumor xenografts. *p < 0.05, **p < 0.01. **(C)** Tumors were harvested on day 12. Tumors from each group were analyzed for the presence of CYP2S1 and p53 by western blotting. GAPDH was used as an internal control. **(D**) Total proteins were extracted and the protein levels of the CYP2S1 were analyzed in Western blot analysis. Results are of the average from three experiments. Data are represented as mean ± s.d., ** p < 0.01. **(E,F**) Weights of xenograft tumours derived from p53+/+ or p53−/−HCT116 cells expressing CYP2S1 knockdown or control (shRNA or shNT, respectively) after oxaliplatin treatment. n = 5 mice per group.

As knockdown of CYP2S1 expression accelerates colorectal tumor growth *in vivo*^25^, we next investigated the effects of oxaliplatin on CYP2S1 protein expression *in vivo*. Isogenic p53+/+ and p53−/−HCT116 cell lines were injected into the flanks of immunocompromised nude mice, and (phosphate-buffered saline) PBS or oxaliplatin treatment was started when the tumors measured approximately 100 mm^3^. Tumor growth was monitored every 2 days until the mice were sacrificed on day 12, when tumors were excised and analyzed by western blotting. Oxaliplatin treatment had a striking antitumor effect on p53+/+HCT116 tumor xenografts (Fig. 5A), whereas the growth of p53−/−HCT116 xenografts was not significantly affected by oxaliplatin treatment (Fig. 5B). Oxaliplatin notably enhanced the induction of CYP2S1 expression in p53+/+HCT116 tumors but had virtually no effect on CYP2S1 expression in p53−/− HCT116 tumors (Fig. 5C,D).

To further examine the effects of CYP2S1 in regulation tumor growth after oxaliplatin treatment *in vivo*, we proceeded xenograft experiments using of CYP2S1 knockdown p53+/+ and p53−/−HCT116 cells. Except for slightly increased cell proliferation, no significant changes in their sensitivity to oxaliplatin were observed in either CYP2S1 shNT or CYP2S1 shRNA HCT116 cells with deletion of p53. By contrast, knockdown of CYP2S1 markedly resisted these cells to oxaliplatin in p53+/+ HCT116 cells (Fig. 5E,F). These observations indicate that the increase in oxaliplatin sensitivity due to CYP2S1 upregulation is associated with p53 expression in colorectal tumor growth *in vivo*.

## Discussion

Oxaliplatin-based neoadjuvant chemotherapy has been used for the past decade as the first-line curative option for CRC patients. Breakthrough research from China has indicated that oxaliplatin can cure advanced primary liver cancer and enhance systemic immunity^38^. However, drug metabolism in target tissues, which is dependent on transporters and metabolic enzymes, may affect the efficacy of oxaliplatin treatment. Many genes related to the effects of oxaliplatin treatment in digestive system cancers have been identified. For instance, overexpression of the organic anion-transporting polypeptide OATP1B3 confers an antiapoptotic effect that counteracts oxaliplatin in colon cancer cells, and this effect may be associated with the transporter activity of OATP1B3^39^. The Bmal1 gene, a core component of the circadian clock in mammals, is involved in the effect of oxaliplatin on CRC, possibly because the circadian system controls drug metabolism and the expression of drug targets as well as cell-cycle progression^40^. However, the mechanism by which CYP450 genes influence the effects of oxaliplatin treatment remains to be fully elucidated.

CYP450 proteins are monooxygenases that catalyze many reactions involved in drug metabolism. In addition to the well-established role of p53 in preventing tumorigenesis, p53 mutations have been associated with reduced effectiveness of chemotherapy^41^. p53 regulates several CYP450 genes, including CYP3A4, CYP3A7, CYP4F2, CYP4F3, CYP19A1, CYP24A1, and CYP21A2^15^,^42^, which are involved in various molecular metabolic pathways. In the present study, we analyzed the impact of p53 status on the induction of CYP2S1 expression in response to oxaliplatin treatment in CRC cells. Treat with oxaliplatin upregulated CYP2S1 expression in a CRC cell line expressing wild-type p53 but not in cell lines lacking p53. The antitumor activity of oxaliplatin in a nude mouse xenograft tumor model also indicated tumor growth to be substantially suppressed in wild-type p53-expressing CRC cells, whereas little effect of oxaliplatin was observed in p53-deficient cells. CYP2S1 knockdown cells conferred a survival advantage after oxaliplatin treatment to cells harboring wild-type p53 *in vitro* and *in vivo*. Ectopic expression p53 results indicated that oxaliplatin treatment can increase CYP2S1 transcriptional activity. These results suggest that p53 is critical for inducing CYP2S1 gene expression in response to oxaliplatin in CRC cells. Thus, the regulation of cytochrome P450 enzymes by p53 may influence drug dosage and drug efficacy.

Wnt/β-catenin signaling is important in cancer tumorigenesis, prognosis, and therapy resistance^26^,^43^, and deregulation of β-catenin can induce p53 activation^31^. Interestingly, activation of p53 by either genotoxic stress or its overexpression can induce a feedback loop that leads to increased degradation of β-catenin in a variety of cell types^37^. A recent bioinformatic analysis also suggested that expression of Wnt/β-catenin pathway genes is correlated with the effectiveness of oxaliplatin treatment in CRC cell lines^24^. Indeed, oxaliplatin significantly inhibited cell growth and decreased β-catenin expression in p53+/+ HCT116 cells. Moreover, treatment of HCT116 cells with oxaliplatin resulted in a reduction in prostaglandin E2 (PGE2) production. Exogenous PGE2 or overexpression β-catenin led to a significant cell survival advantage after oxaliplatin treatment. As the TOPFlash/FOPFlash reporter activity assay indicated that oxaliplatin downregulates Wnt signaling. CRC cell growth inhibition due to oxaliplatin may be partially dependent on the cell cycle.

**Figure 6:**
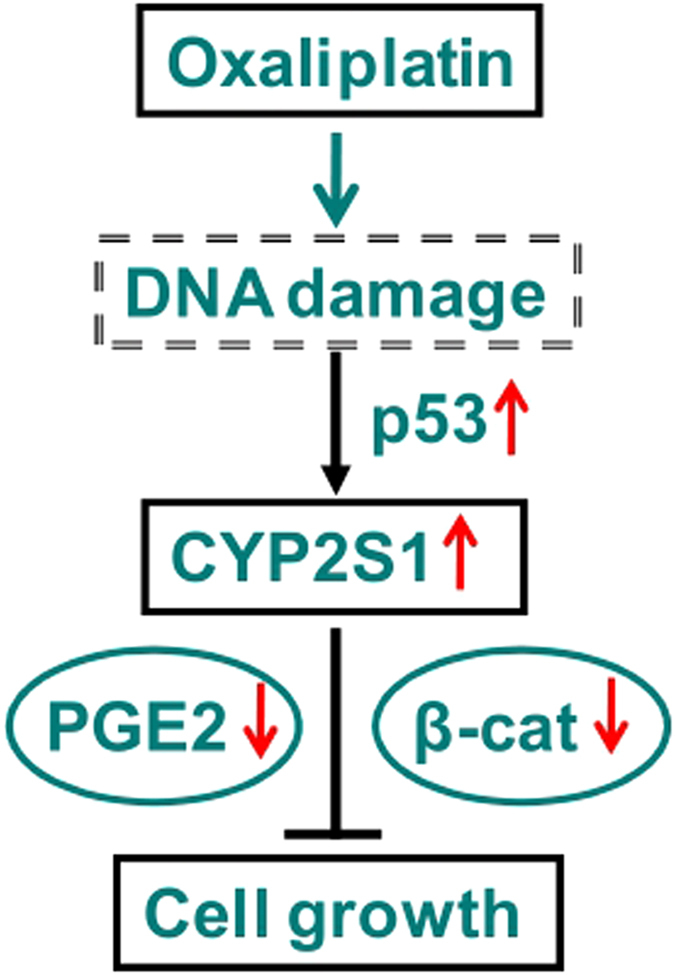
Inhibition of the growth of colorectal tumor cells by oxaliplatin treatment. When CRC cells are exposed to oxaliplatin, activation of p53 leads to upregulation of CYP2S1 transcription and translation and a decrease in endogenous PGE2 biosynthesis and β-catenin expression, which ultimately decreases colorectal cell survival and proliferation.

In conclusion, our results demonstrate that oxaliplatin significantly inhibits CRC cell growth and that this inhibition may be associated with simultaneous increases in CYP2S1 and p53 expression, leading to downregulation of Wnt/β-catenin signaling (Fig. 6). Our results provide potential biomarkers of chemotherapeutic sensitivity in CRC cell lines. Future studies are needed to evaluate the full potential of targeting CYP450 genes to enhance the effectiveness of chemotherapy.

## Materials and Methods

### Cell, plasmids, and treatment

Human CRC cell lines SW480 and HT29 were obtained from American Type Culture Collection (Manassas, VA, USA).The isogenic p53+/+(wild-type p53) HCT116 and p53−/− HCT116 cell lines were kind gifts from Dr. Xuemei Tong. Cells were cultured in RPMI-1640 medium with 2.5 mM glutamine supplemented with 10% fetal bovine serum and 1% penicillin/streptomycin solution at 37 °C in a humidified atmosphere with 5% CO_2_. Oxaliplatin (Sigma; St. Louis, MO, USA) at the indicated μM concentrations was added for different time periods to the culture medium of exponentially growing cells. The expression plasmid for p53 (wild-type) was kind gifts from Dr. Xuemei Tong. β-catenin plasmids (kindly provided by Prof. Lin Li). Transfection was transfected using Lipofectamine 2000 (Gibco 11668-027) according to the manufacturer’s instructions.

### Cell proliferation assays

Cell proliferation was determined using a Cell Counting Kit-8 assay (CCK8, Sigma, lot#BCBK6742V), according to the manufacturer’s instructions. In brief, cells were seeded in 96-well plates at a density of 4 × 10^3^cells per well in 100 μl growth medium and allowed to adhere overnight. After an attachment period of 24 h, the cells were treated with different concentrations of oxaliplatin (μM) for different time periods. At the end of treatment, 10 μl of tetrazolium substrate was added to each well of the plate. The plates were incubated at 37 °C for 1 h, and the optical density (OD) at 450 nm was then measured using a Bio-Rad ELISA microplate reader (Bio-Rad 680, Japan).

### CYP2S1 short hairpin RNA transfection

Cells were grown on a 6-well plate (1 × 10^5^ cells per well) for 24 h and transfected with human-specific CYP2S1 short hairpin RNA (shRNA) plasmids (Genechem shRNA, NM_030622) using Lipofectamine 2000 (Gibco 11668-027) following the manufacturer’s instructions. The nontargeting was used as a control (shNT). The culture was selected with 1ug/ml puromycin (Sigma P8833; Sigma-Aldrich) for 4 days and then 0.5 ug/ml for three weeks. CYP2S1 mRNA and protein expression was analyzed using quantitative real-time polymerase chain reaction (qRT-PCR) and Western blot analysis.

### Western blotting

Cells (10^6^/well) were seeded in six-well plates and treated with the indicated concentrations of oxaliplatin (μM) or PBS for 24 h. The cells were then rinsed with PBS and lysed using 1ml of lysis buffer (consisting of 40 mMTris-Cl, 2 Mthiourea, 7 Murea, 4% CHAPS and 5 μl of cocktail protease inhibitor) for 60 min on ice. The samples were maintained on ice and sonicated intermittently. Proteins were quantified using the Bradford method according to the manufacturer’s instructions and frozen at −80 °C until analysis. Antibodies against CYP2S1 (Abgent, AP12635b), GAPDH (Santa Cruz, SC-365062), β-catenin(CST 9587), p63(Abcam, ab124762), p73(Abcam, ab40658) and p53(DO-1; Santa Cruz, SC-126) were used. Equal amounts of protein (30 μg) were separated by sodium dodecyl sulfate-polyacrylamide gel electrophoresis (SDS-PAGE) (10% separating gel). After electrophoresis, the proteins were transferred to PVDF nitrocellulose membranes (Santa Cruz Biotechnology, Sc-3723, Lot#B2703). The blots were then blocked in 5% nonfat dry milk solution for 1 h at room temperature. The membranes were incubated with the respective primary antibodies at room temperature, followed by anti-rabbit or anti-mouse alkaline phosphatase conjugated secondary IgG antibodies. Finally, the blots were incubated with ECL chemiluminescent reagent (Santa Cruz Biotechnology, Paso Robles, California) and exposed to X-ray film.

### Reverse-transcription and quantitative real-time PCR

Cells (10^6^/well) were seeded in six-well plates and treated with the indicated concentrations of oxaliplatin (μM) or PBS for 24 h. The cells were then rinsed with PBS. Total RNA was extracted using the TRIzol method (Life Technologies, CA), followed by gel electrophoresis as well as NanoDrop spectrometric analysis (NanoDrop 2000 Spectrophotometer, Thermo Scientific) for quality control. A 2-μg aliquot of RNA was used for complementary DNA (cDNA) synthesis in a total reaction volume of 20 μl with the PrimeScript RT Reagent kit (Takara Bio, Dalian, China). Quantitative PCR was performed using Taqman Gene Expression Assays (Applied Biosystems) and a ViiA 7 instrument (Life Technologies, CA), with four replicates. GAPDH mRNA was quantified as the endogenous control to normalize gene expression.

### PGE2 immunoassay for quantification of PGE2

The levels of PGE2 in cell homogenates were determined using Cayman Chemicals PGE2 Enzyme Immunoassay Kit. Briefly, cells were harvested at the desired time point, homogenized in 100 mM phosphate buffer, pH 7.2, and subjected to repeated freeze-thaw cycles to release the intracellular components. The PGE2 concentration of the supernatants was determined according to the manufacturer’s instructions, and normalized to the total protein content. The PGE2 concentration is presented as picograms of PGE2 per microgram of total protein. Statistical analysis was performed using Student’s t test.

### Treatment of cells with exogenous PGE2

Cells were plated onto 6-well plates (2 × 10^5^ cells/mL) and were serum deprived for 24 hr and then stimulated with 50 nM PGE2 (Sigma Aldrich, P0409) and oxaliplatin (20 μM) for 24 h. Cells were collected and RNA was extracted for qRT-PCR.

### Luciferase reporter assays

Cells (2 × 10^5^/well) were plated in 6-well plates. After 24 h, 200ng of TOPFlash or FOPFlash and 200ng of EGFP-C1 plasmids (kindly provided by Prof. Lin Li) were co-transfected using Lipofectamine 2000 (Gibco 11668-027) and basal medium (RPMI-1640 without antibiotics and FBS). Six hours after transfection, the basal medium was aspirated, and 20 μM oxaliplatin in complete RPMI-1640 medium was added. After 24 h, the cells were lysed and assayed using the Luciferase RGA high sensitive kit (Roche). The reporter-driven luciferase activity was quantified by luminescence collection using a Synergen 2 (BioTek) reader. Luciferase activity was normalized to GFP expression levels as previously described^44^. The TOP/FOP ratio was used as a measurement of TCF/LEF-mediated transcriptional activity. Average activity and standard deviations were calculated from three independent experiments.

### Xenograft experiments

All mouse experiments were approved by the Animal Care Committee of the University of SJTU and were performed according to Bio-X institutional guidelines. Briefly, four-week-old athymic nude mice received flank injections of 2 × 10^6^ p53+/+HCT116 or p53−/−HCT116 (or CYP2S1 shNT, CYP2S1 shRNA) cells resuspended in 200 μl PBS. Tumors were allowed to grow for 10 days until a volume of approximately 100 mm^3^ was reached. At day 10, the mice were injected intraperitoneally (i.p.) twice weekly with 10mg/kg oxaliplatin. Mice that did not receive oxaliplatin received equivalent injections of PBS. The tumor sizes were measured every 2 days with a caliper, and tumor volume (mm^3^) was determined as V = short diameter^2^ × long diameter × 0.5. The mice were sacrificed on day 22 after tumor cell injection. The tumors in each group were excised to examine protein expression by western blotting.

### Statistical analysis

Results are expressed as the means ± SD. Statistical significance (P values) between the control and experimental groups was calculated using Student’s t-test.

## Additional Information

**How to cite this article**: Yang, C. *et al.* Upregulation of CYP2S1 by oxaliplatin is associated with p53 status in colorectal cancer cell lines. *Sci. Rep.* **6**, 33078; doi: 10.1038/srep33078 (2016).
